# Uncinariasis

**Published:** 1907-03

**Authors:** 


### EDITORIAL NOTES AND COMMENTS.

The Business Office of the JOURNAL-RECORD is 1007-1008 Century Building.

The Editorial Office is 318-319-320 The Prudential.

Address all Business Commucications to Dr. M. B. Hutchins, Manager.

Make remittances payable to THE ATLANTA JOURNAL-RECORD OF MEDICINE.

On matters pertaining to the Editorial and Original communications, address Dr, Bernard Wolff, Atlanta.

Reprints of original articles will be furnished at cost price. Requests for the same should always be made on THE MANUSCRIPT.

We will present, postpaid, on reqnest, to each contributor of an original article, twenty 20) marked copies of THE JOURNAL-RECORD containing such article.

### UNCINARIASIS.

Southern writers have been calling attention more and more to the presence of uncinariasis or hook-worm disease in the South. Among these Drs. H. F. Harris and Claude A. Smith have done much original and pioneer work in investigating the disease. It is found to be prevalent in the State of Georgia especially in the mountainous portions of the State. When developed the disease conspicuously stamps its victim. The individual affected has a look of presenility, the facies is that of a person suffering from marked debility, the mucous membranes are pallid, the skin sallow, the musculature underdeveloped, the body stunted in growth. Anaemia is profound. Spontaneous recovery sometimes occurs, but as a rule the patient succumbs to exhaustion after the disease has lasted for a period varying within wide limits, sometimes as much as twenty years. The parasite enters the skin and produces a dermatitis known as ground itch, dew poison, toe itch. The worm finally finds its way into the intestinal canal where it makes its home attaching itself to the wall of the gut by means of hooklets. The eggs of the female do not hatch in the bowel, but require extracorporeal development. The eggs may be found in the feces by microscopic examination and the mature parasites are visible to the naked eye in the stools when diffused in water.

The disease appears to be readily curable by large doses of thymol administered in very much the same manner as in the treatment of amoebic dysentery.

It is well for practitioners in districts where uncinariasis is known to exist, to be on the lookout for the disease and in suspected cases examine the feces or send them to the State Board of Health.

